# Acute effect of high-intensity interval exercise on vascular endothelial function and possible mechanisms of wall shear stress in young obese males

**DOI:** 10.3389/fphys.2022.966561

**Published:** 2022-09-16

**Authors:** Wenxia Shi, Haibin Liu, Ling Cao, Yufeng He, Pei Su, Jiangang Chen, Mengyue Wang, Xulong Li, Shuang Bai, Donghui Tang

**Affiliations:** ^1^ College of P.E. and Sport, Beijing Normal University, Beijing, China; ^2^ School of Kinesiology and Health Promotion, Dalian University of Technology, Dalian, China; ^3^ Sinopec Research Institute of Petroleum Processing, Beijing, China; ^4^ Department of P.E., Qingdao University of Technology, Qingdao, China; ^5^ Capital Institute of Physical Education and Sports, Beijing, China

**Keywords:** high-intensity interval exercise, young obese males, vascular endothelial function, wall shear stress, inflation

## Abstract

**Objective:** To investigate the mechanisms of wall shear stress (WSS) responsible for the effects of high-intensity interval exercise (HIIE) on vascular endothelial function in young obese males.

**Methods:** A within-subject study design was used. We examined the response of the reactive hyperemia index (RHI) to acute HIIE in young obese males (*n* = 20, age = 20.38 ± 1.40 years, body mass index [BMI] = 31.22 ± 3.57, body fat percentage [BF (%)] = 31.76 ± 3.57). WSS was manipulated using 100, 80, or 60 mmHg cuff inflation during the HIIE to determine the proper inflation capable of maintaining WSS near baseline levels. One-way repeated measures analysis of variance and LSD post hoc tests were performed to compare changes in WSS and vascular endothelial function at baseline HIIE and following HIIE using different cuff inflations.

**Results:** There were no significant differences in RHI and WSS between the three cuff inflation values (*p* > 0.05). WSS was significantly higher in obese male individuals after HIIE and HIIE with 100 mmHg cuff inflation (*p* = 0.018, *p* = 0.005) than that at baseline, with no significant differences observed comparing HIIE and HIIE with 100 mmHg inflation (*p* = 0.23). The RHI after HIIE was significantly higher (*p* = 0.012) than that at baseline, while no significant differences were detected after HIIE at 100 mmHg (*p* = 0.91). The RHI was significantly lower after HIIE with 100 mmHg than that after HIIE (*p* = 0.007). WSS (*p* = 0.004) and RHI (*p =* 0.017) were significantly higher after HIIE than that at baseline, while no significant differences were observed after HIIE with either 80 or 60 mmHg cuff inflation (baseline vs. HIIE + 80 mmHg: WSS: *p* = 0.33, RHI: *p* = 0.38; baseline vs. HIIE + 60 mmHg: WSS: *p* = 0.58, RHI: *p* = 0.45). WSS was similar to HIIE, after HIIE with either 80 or 60 mmHg inflation (*p* = 0.36, *p* = 0.40). However, RHI was significantly higher for HIIE than for HIIE with both 80 and 60 mmHg inflation (*p* = 0.011, *p* = 0.006).

**Conclusion:** HIIE could significantly improve WSS and vascular endothelial function. HIIE intervention with 60 or 80 mmHg inflation might enhance WSS near the baseline level. HIIE-induced acute changes in WSS may provide the primary physiological stimulus for vascular endothelial adaptation to HIIE in young obese males.

## 1 Introduction

Epidemiological studies have found that obesity in adolescence can predict a series of future health problems, and is an independent risk factor for the development of hypertension, diabetes, and atherosclerosis-related diseases in adulthood (Lavie et al., 2014; Weihrauch-Blueher et al., 2019). Recent research has shown that vascular endothelial dysfunction occurs during the initial stage of atherosclerosis (Mudau et al., 2012), and severe obesity is closely related to early vascular endothelial dysfunction (Avogaro and de Kreutzenberg, 2005; Kwaifa et al., 2020). Alterations in wall shear stress (WSS) have an extremely important effect on vascular endothelial dysfunction (Souilhol et al., 2020; Chistiakov et al., 2017).

WSS is a friction force that is exerted parallel to the inner wall of the blood vessel and effectively promotes endothelial cytokine release and inhibits inflammation and oxidative stress. WSS can also promote a healthy state of the vascular endothelium while maintaining its normal direction and size (Zhou et al., 2014; Souilhol et al., 2020; Theofilis et al., 2021). Currently, it is generally believed that a higher WSS within a certain range is conducive to maintaining normal vascular endothelial function (Baeyens et al., 2016). Furthermore, the vascular endothelium is damaged or prone to thrombosis under extremely high WSS conditions, which could promote the appearance and development of atherosclerotic plaques (Green et al., 2017). To some extent, excessive fat accumulation and arterial hypertrophy in obese people may lead to below-normal WSS (Hamburg et al., 2010; Slattery et al., 2016). Therefore, we can speculate that a lower WSS could be an important cause of vascular endothelial dysfunction in obese individuals.

Regular exercise is an important means of improving vascular endothelial function in obese individuals. Aerobic exercise, aerobic exercise combined with resistance exercise, and aerobic exercise combined with diet control may effectively improve vascular endothelial dysfunction in obese adolescents (Li and Chen, 2013; Tang and Bai, 2017; Wang et al., 2018). However, for obese adolescents, a lack of time and interest are the main obstacles restricting their participation in moderate-intensity continuous training. Recently, high-intensity interval training (HIIT) has gradually become an important exercise program for overweight and obese people to promote weight loss because of its short training time, high benefits, and enjoyment (Bartlett et al., 2011). Furthermore, some studies have verified that HIIT can effectively enhance vascular endothelial function in obese people (Sawyer et al., 2016; Chuensiri et al., 2018; Fernandes et al., 2020). However, the mechanism by which HIIT improves vascular endothelial function is mainly attributed to its improvement in traditional risk factors for cardiovascular disease. Thijssen et al. (2009) found that WSS played a key role in mediating vascular adaptation in a series of human studies using a unilateral model combined with cuff inflation to attenuate WSS during different exercises, including bilateral handgrip exercise training and cycle exercise (Thijssen et al., 2009; Thijssen et al., 2011). These studies also provided information on the systemic effect of exercise training through mechanisms dependent on WSS (Birk et al., 2012; Green et al., 2017; Chuensiri et al., 2018). However, to our knowledge, these studies were conducted on young healthy male individuals, but no study on individuals with obesity has been conducted. Furthermore, little attention has been paid to the mechanism of WSS in HIIE to improve vascular endothelial function in obese individuals.

The primary objective of this study was to explore the mechanism of WSS following HIIE to improve vascular endothelial function in young obese males. In the present study, we manipulated WSS in obese male individuals locally using 3 different cuff inflations (100, 80, and 60 mmHg) to determine the inflation that could maintain WSS near baseline levels after HIIE exercise. Vascular endothelial function was also measured after acute HIIE with different inflations to determine the appropriate pressure. Thus, by comparing HIIE with the proper inflation, resting state (or baseline), and HIIE, we hypothesized that compared with baseline, WSS and RHI would be significantly higher after HIIE, and WSS and RHI after HIIE with proper pressure would be close to baseline values. Furthermore, we predicted that improvement in vascular endothelial function after acute HIIE could be related to the brachial artery being exposed to greater WSS during HIIE training.

## 2 Materials and methods

### 2.1 Participants

After screening, 20 male obese students were recruited from a local college. The inclusion criteria were as follows: 1) age: 18–22 years old; 2) BMI ≥ 28 kg/m^2^; and 3) low physical activity level as assessed by International Physical Activity Questionnaire (IPAQ), with insufficient activity (<150 min/week or <600 MET/week). Individuals with a history of cardiovascular, endocrine and/or metabolic diseases were excluded. Furthermore, the Physical Assessment Risk Questionnaire (PAR-Q) was used to determine the risks of participants engaging in exercise (Table 1). The sample size calculation was conducted by *a priori* analysis (G*Power 3.1.9.7, Dusseldorf, Germany) based on repeated measures analysis of variance (ANOVA) within factors, with power (1-βerr prob) = 0.8, alpha level = 0.05, and effect size = 0.33, using the data provided in a previous study evaluating physiological adaptations to high-intensity interval training combined with blood flow restriction in master road cyclists (Tangchaisuriya et al., 2021). A total sample size of 14 participants was estimated. To avoid loss of sample size, we increased the number of participants to 20. Basic information about the participants is shown in Table 1. Participants voluntarily provided their written informed consent after understanding the experimental process and purpose. This experimental procedure was approved by the Ethics Committee of the College of Physical Education and Sports, Beijing Normal University.

**TABLE 1:** Basic information of the participants (*n* = 20).

	Mean ± SD
Age (years)	20.38 ± 1.40
Height (cm)	175.79 ± 6.29
Weight (kg)	96.73 ± 14.47
BMI (kg/m^2^)	31.22 ± 3.57
Body fat (%)	31.76 ± 3.57
WC (cm)	99 ± 10.59
NC (cm)	40.31 ± 1.65
Fat mass (kg)	30.73 ± 9.63
Muscle mass (kg)	62.29 ± 6.03
BM (kcal)	1966 ± 213
TEM (kcal)	2919 ± 316
SBP (mm/Hg)	123.24 ± 10.89
DBP (mm/Hg)	74.42 ± 6.13
HR (bpm)	70.19 ± 11.31

Note: FM, fat content; MM, muscle content; BF (%), percentage of body fat; WC, waist circumference; NC, neck circumference; BM, basic metabolism; TEM, total energy metabolism.

### 2.2 Experimental design

**FIGURE 1:**
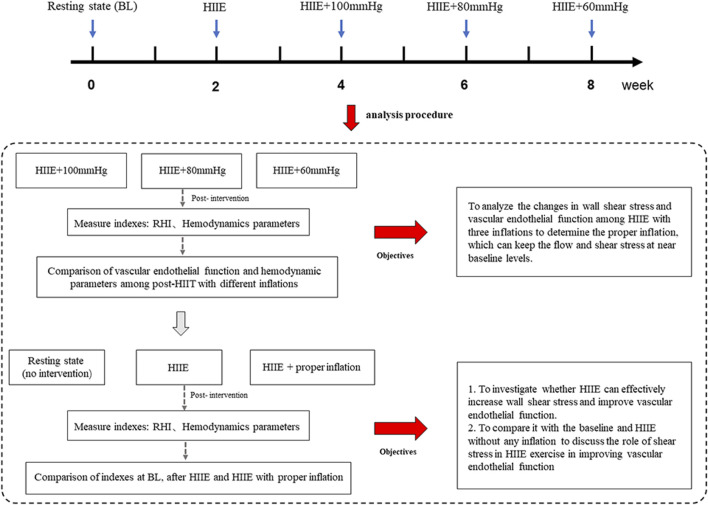
The experimental design. Note: HIIE + 100 mmHg, HIIE with 100 mmHg inflation; HIIE + 80 mmHg, HIIE with 80 mmHg inflation; HIIE + 60 mmHg, HIIE with 60 mmHg inflation; RHI, reactive hyperemia index.

A self-controlled experimental design was adopted, which involved a within-subject design. In this study, only one group (*n* = 20) was included and five tests on vascular endothelial function and hemodynamic parameters were performed, including at baseline (resting state without intervention), after HIIE, post-HIIE with 100 mmHg inflation, post-HIIE with 80 mmHg inflation, and post-HIIE with 60 mmHg inflation. Meanwhile, there was a 2-week washout period between each test. This study aimed to investigate whether HIIE could effectively increase WSS and improve vascular endothelial function. Further, the study analyzed the changes in WSS and vascular endothelial function during HIIE with three cuff inflations to determine the appropriate inflation to keep WSS near the baseline level. On this basis, we further compared HIIE with proper inflation, at baseline and following HIIE to evaluate the role of WSS in HIIE in improving vascular endothelial function, as shown in Figure 1.

### 2.3 Acute high-intensity interval exercise exercise intervention program

Thijssen et al. (2009) conducted a series of in-depth studies on WSS mechanisms by which exercise improved vascular endothelial function (Thijssen et al., 2009; Thijssen et al., 2011). Among them, comparing the shear stress stimuli that existed in the cuffed versus uncuffed arms during a bilateral handgrip exercise, they found that the 8-week bilateral handgrip exercise could increase WSS and improve vascular endothelial function in the uncuffed arm, but the cuffed arm did not show any significant changes. They also found that rhythmic lower-limb exercise (cycling and walking) could increase the shear rate in a subsequent study (Thijssen et al., 2009; Tinken et al., 2009; Birk et al., 2012; Atkinson et al., 2015). Considering the peculiarity of obese youth, we chose cycling exercise for HIIE to avoid injury to the knee joint or ankle joint due to the large load of body weight. In the present study, we extended the cycle exercise data in obese men from that experiment to further enrich the types of exercise and the groups.

**FIGURE 2:**
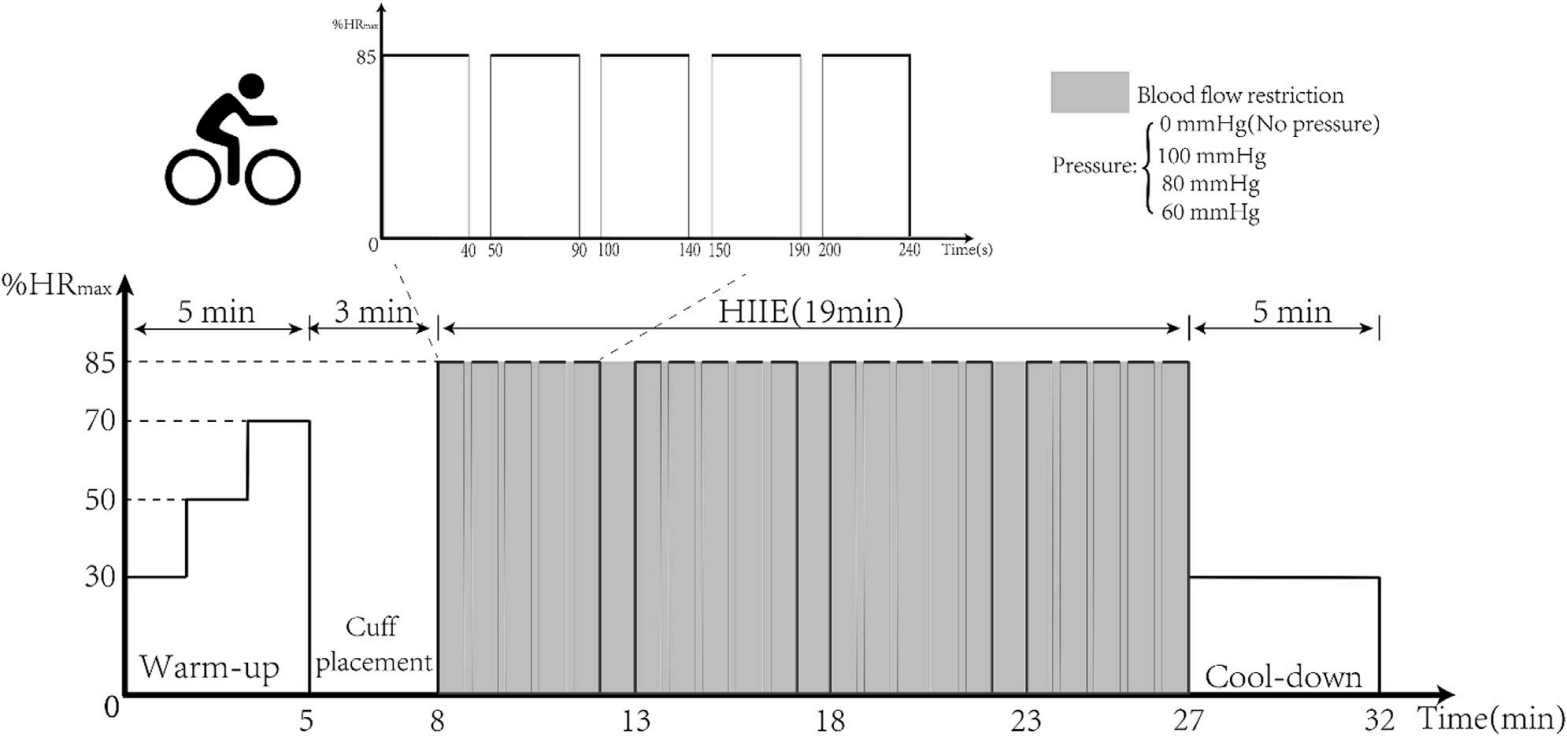
HIIE intervention with different inflations.

First, baseline vascular endothelial function and hemodynamic indices were tested using EndoPAT and high-resolution Doppler ultrasound. Then HIIE was performed for 16 min and was carried out using aerobic power bicycles (Powermax-VIII, Combi Wellness, Japan) (Ramos et al., 2015). The intensity of exercise was 85%–95% of the maximum heart rate (HRmax), which was calculated indirectly using the formula (220–age). The HIIE intervention was set up with three groups, and the first group performed five sets of HIIE using a 3-kp load for 40 s of exercise and 10 s of rest. With adaptation to intensity, in the second and third groups, the same exercise was performed with a 4-kp load. Standard cuffs were worn at 2 cm from the brachial artery of the upper limb and inflated to 100, 80, and 60 mmHg (BFR BRANDS, United States) before HIIE, as shown in Figure 2. After the 16-min HIIE intervention with different inflations, the reactive hyperemia index (RHI) and hemodynamic parameters were examined immediately.

### 2.4 Experimental measures

#### 2.4.1 Vascular endothelial function

The participant was placed in a supine position on the test bed in a quiet state and the EndoPAT2000 biosensor (Itamar Medical Co., Ltd., Israel) was placed at the front of the index finger of both hands, the standard cuff was worn at 2 cm from the brachial artery of the nondominant arm, and the procedure was started. Initially, the baseline tension data were collected for 6 min. The cuff was then inflated and data were collected 5 min after the blood flow from the brachial artery was blocked. Finally, the ratio of signal amplitude before and after blocking was calculated and corrected by Endo-PAT software, and the RHI was obtained. PAT is a finger plethysmographic device that allows the isolated detection of changes in pulsatile arterial volume (Gerhard-Herman et al., 2002; Bonetti et al., 2004). The higher the RHI value, the better the vascular endothelial function and vice versa. According to the guidelines of the endothelial function test, prior to the test, participants were required to fast for 4 h without the intake of coffee, tea, or sugary foods. Taking medication on the morning of the test day and staying up late was also prohibited. No strenuous exercise was allowed the day before the test; and if any was reported, the intervention and the test would be postponed.

#### 2.4.2 Hemodynamics

Brachial artery diameter and axial blood velocity waveform were measured with a high-resolution Doppler ultrasound (Prosound Alpha 7, Aloka, Japan) from 5 to 7 p.m. First, participants were asked to rest in the supine position for 10 min. Second, the position of the brachial artery was determined by transverse scanning. Third, the transverse section of the brachial artery was longitudinally scanned to obtain pictures of the brachial artery diameter waveform and the axial blood velocity waveform. At the same time, the brachial systolic blood pressure (SBP), diastolic blood pressure (DBP), and heart rate (HR) on the left upper arm were measured using an electronic sphygmomanometer (Patient Monitor PM8000, Mindray). Based on the test data and imaging information, MATLAB (R2011b) software and Womersley theory programming were used to calculate artery diameter (AD), blood velocity (BV), WSS, and the oscillatory shear index (OSI). To avoid the influence of other factors, all participants were not allowed to perform any strenuous exercise before the intervention.

#### 2.5 Statistical analysis

In this study, SPSS 24.0 was used for data processing and analysis. All data are expressed as the mean ± standard deviation (M ± SD), and GraphPad Prism Software 8.0 was used for graphing. Box plots were constructed to visualize the data. Meanwhile, the extreme outliers were defined as data points that were 3 SDs from the mean and were eliminated from the data analyses. The K-S test was performed to examine the normal distribution. One-way repeated measures analysis of variance and the LSD post hoc test were used to analyze the data among three tests. *p-*values < 0.05 were considered statistically significant.

## 3 Results

### 3.1 Changes in reactive hyperemia index and wall shear stress after high-intensity interval exercise intervention with different cuff inflations

The results of the one-way repeated measures analysis of variance showed that WSS after HIIE with 100 mmHg inflation was significantly higher than that after HIIE with 80 mmHg inflation (HIIE + 100 mmHg vs. HIIE + 80 mmHg: 1.02 ± 0.23 vs. 0.83 ± 0.17, *p* = 0.042), and there was no significant difference in the change in WSS following the other interventions (HIIE + 60 mmHg vs. HIIE + 80 mmHg: 0.90 ± 0.17 vs. 0.83 ± 0.17, *p* = 0.20; HIIE + 60 mmHg vs. HIIE + 100 mmHg: 0.90 ± 0.17 vs. 1.02 ± 0.23, *p* = 0.24).

**FIGURE 3:**
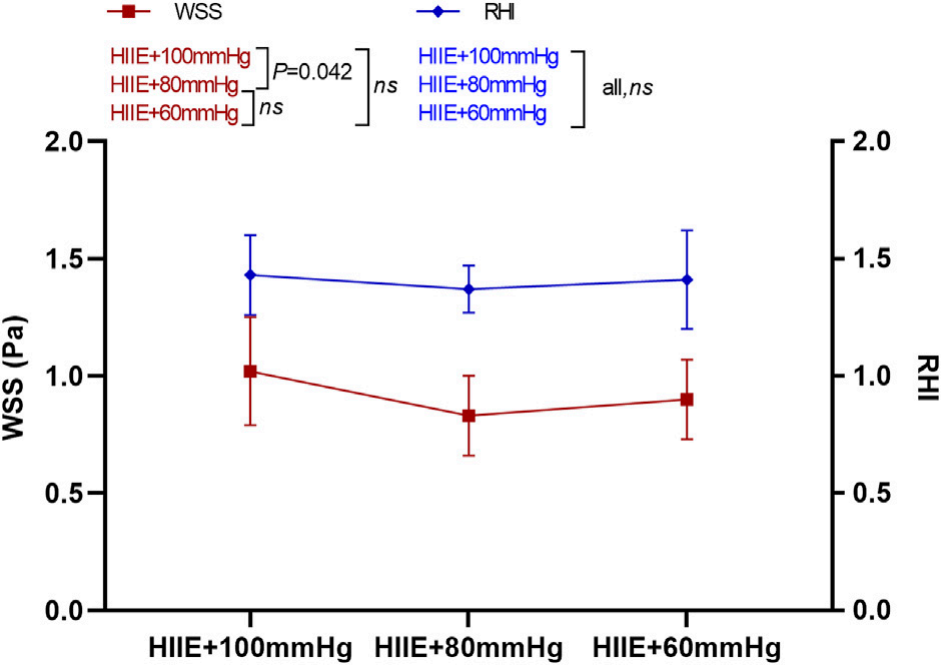
Changes in RHI and WSS after HIIE intervention with different inflations. Note: RHI, reactive hyperemia index; WSS, wall shear stress.

RHI did not change significantly across HIIE with different cuff inflations (HIIE + 100 mmHg vs. HIIE + 80 mmHg: 1.43 ± 0.17 vs. 1.37 ± 0.10, *p* = 0.27; HIIE + 80 mmHg vs. HIIE with 60 mmHg: 1.37 ± 0.10 vs. 1.41 ± 0.21, *p* = 0.51; or HIIE + 60 mmHg vs. HIIE+100 mmHg: 1.41 ± 0.21 vs. 1.43 ± 0.17, *p* = 0.76) (Figure 3).

### 3.2 Changes in reactive hyperemia index and wall shear stress at baseline and after high-intensity interval exercise and high-intensity interval exercise with 100 mmHg inflation

The results of a one-way repeated measures ANOVA showed that compared with baseline, HIIE and HIIE with inflation of 100 mmHg induced a significant increase in WSS (baseline vs. HIIE: 0.77 ± 0.20 vs. 0.91 ± 0.26, *p* = 0.018; baseline vs. HIIE + 100 mmHg: 0.77 ± 0.20 vs. 1.03 ± 0.24, *p* = 0.005). However, there was no significant difference in WSS between HIIE and HIIE + 100 mmHg inflation (HIIE vs. HIIE + 100 mmHg: 0.91 ± 0.26 vs. 1.03 ± 0.24, *p* = 0.23).

**FIGURE 4:**
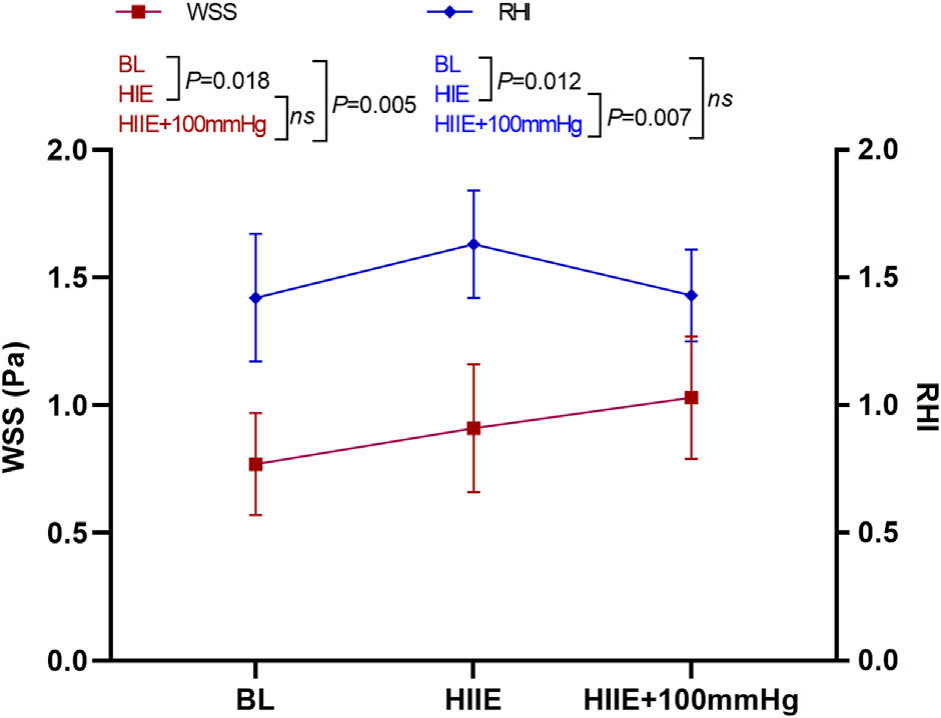
Changes in RHI and WSS at baseline, after HIIE, and after HIIE with 100 mmHg inflation intervention. Note: RHI, reactive hyperemia index; WSS, wall shear stress.

The RHI was significantly higher than that at baseline after the HIIE intervention (baseline vs. HIIE: 1.42 ± 0.25 vs. 1.63 ± 0.21, *p* = 0.012), but acute HIIE with inflation of 100 mmHg did not change the RHI (baseline vs. HIIE + 100 mmHg: 1.42 ± 0.25 vs. 1.43 ± 0.18, *p* = 0.91). The RHI following HIIE with 100 mmHg inflation was significantly lower than that of HIIE (HIIE vs. HIIE + 100 mmHg: 1.63 ± 0.21 vs. 1.43 ± 0.18, *p* = 0.007) (Figure 4).

### 3.3 Changes in reactive hyperemia index and wall shear stress at baseline, after high-intensity interval exercise and high-intensity interval exercise with 80 mmHg inflation

WSS was significantly higher than that at baseline after the HIIE intervention (baseline vs. HIIE: 0.80 ± 0.20 vs. 0.97 ± 0.24, *p* = 0.004), but the change in WSS after HIIE with 80 mmHg inflation was not statistically significant (baseline vs. HIIE + 80 mmHg: 0.80 ± 0.20 vs. 0.88 ± 0.25, *p* = 0.33). There was also no statistically significant difference between HIIE and HIIE with 80 mmHg inflation (HIIE vs. HIIE + 80 mmHg: 0.97 ± 0.24 vs. 0.88 ± 0.25, *p* = 0.36).

**FIGURE 5:**
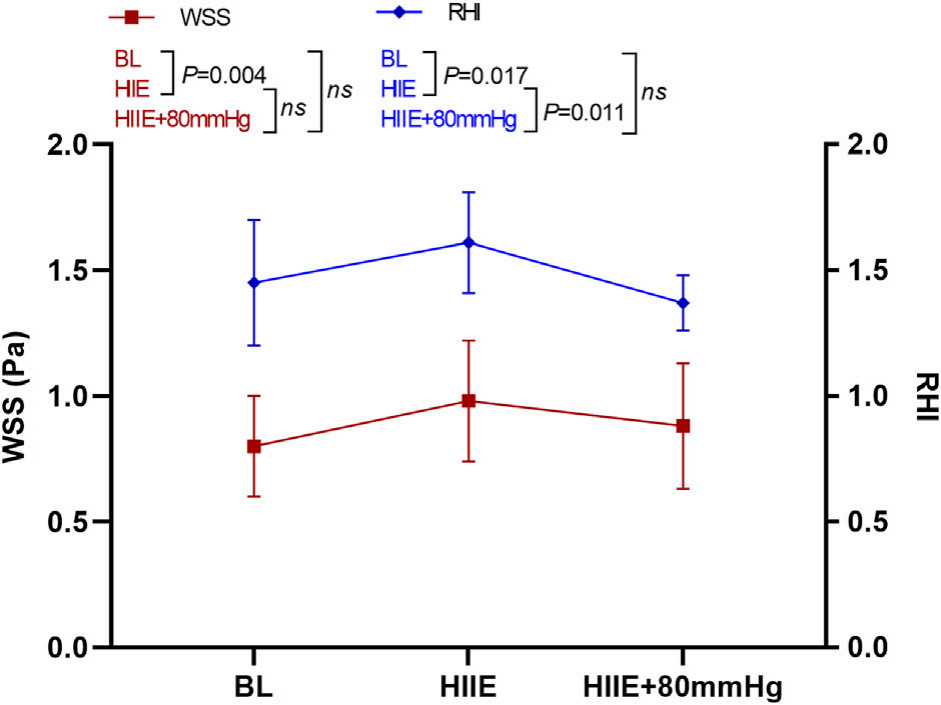
Changes in RHI and WSS at baseline after HIIE and HIIE with 80 mmHg inflation. Note: RHI, reactive hyperemia index; WSS, wall shear stress.

However, compared with baseline, RHI after HIIE increased significantly (baseline vs. HIIE: 1.45 ± 0.25 vs. 1.61 ± 0.20, *p* = 0.017). The RHI after HIIE with 80 mmHg inflation was not significantly different from baseline (baseline vs. HIIE + 80 mmHg: 1.45 ± 0.25 vs. 1.37 ± 0.11, *p* = 0.38). The RHI after HIIE with 80 mmHg inflation was significantly lower than that of HIIE alone (HIIE vs. HIIE + 80 mmHg: 1.61 ± 0.20 vs. 1.37 ± 0.11, *p* = 0.011) (Figure 5).

### 3.4 Changes in reactive hyperemia index and wall shear stress at baseline and after high-intensity interval exercise and high-intensity interval exercise with 60 mmHg inflation

WSS was significantly higher after HIIE than at baseline (baseline vs. HIIE: 0.80 ± 0.21 vs. 0.93 ± 0.25, *p* = 0.033), but after HIIE with 60 mmHg inflation, WSS did not change significantly (baseline vs. HIIE + 60 mmHg: 0.80 ± 0.21 vs. 0.84 ± 0.22, *p* = 0.58). The difference was still not significant after HIIE and HIIE with 60 mmHg inflation (HIIE vs. HIIE + 60 mmHg: 0.93 ± 0.25 vs. 0.84 ± 0.22, *p* = 0.40).

**FIGURE 6:**
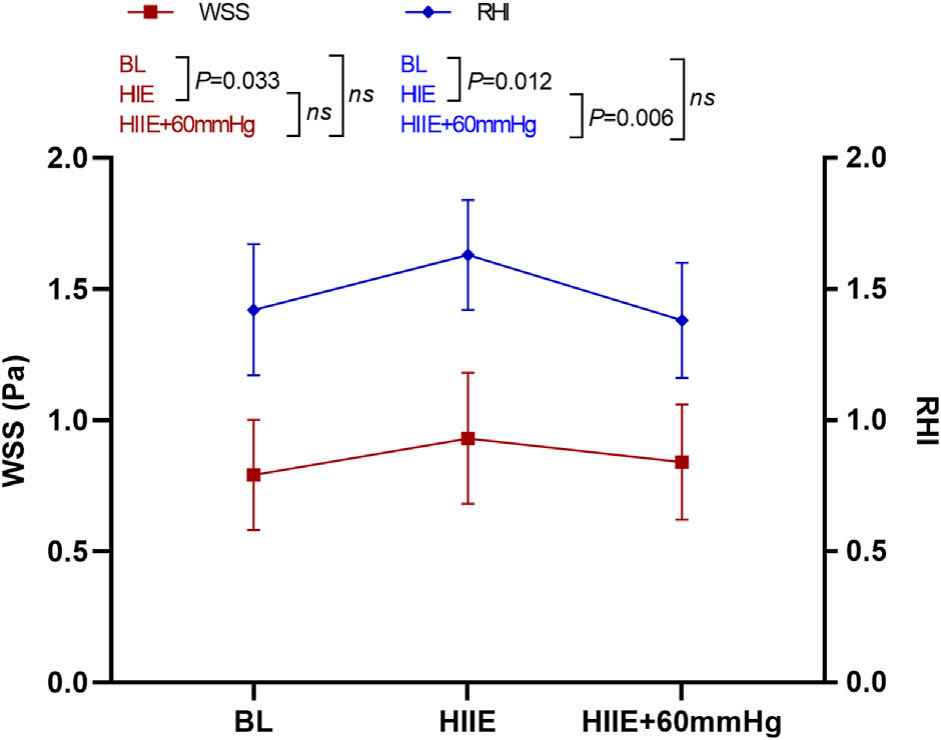
Changes in RHI and WSS at baseline and after HIIE and HIIE with 60 mmHg inflation. Note: RHI, reactive hyperemia index; WSS, wall shear stress.

The RHI was significantly higher after HIIE that at baseline (baseline vs. HIIE: 1.42 ± 0.25 vs. 1.63 ± 0.21, *p* = 0.012), and the RHI after HIIE with 60 mmHg inflation did not differ significantly from baseline (baseline vs. HIIE + 60 mmHg: 1.42 ± 0.25 vs. 1.38 ± 0.22, *p* = 0.45). The RHI was significantly lower in HIIE with 60 mmHg inflation than that following HIIE (HIIE vs. HIIE + 60 mmHg: 1.63 ± 0.21 vs. 1.38 ± 0.22, *p* = 0.006) (Figure 6).

### 3.5 Changes in other hemodynamic parameters after high-intensity interval exercise intervention with different inflations

The one-way repeated measures analysis of variance showed that blood velocity after HIIE and HIIE with 100 mmHg inflation was significantly higher than the baseline levels (*p* < 0.05). Blood velocity after HIIE with 100 mmHg inflation was significantly higher than that following HIIE alone (*p* < 0.05). There was no significant difference in blood velocity after HIIE with 80 and 60 mmHg inflation (*p* > 0.05).

As illustrated in Table 2, there was no significant difference in the changes in artery diameter across the intervention programs (all, *p* > 0.05). However, our results indicated that the OSI in HIIE was significantly lower than the baseline level (*p* < 0.05). Compared with HIIE, the OSI increased significantly after HIIE with 100 and 80 mmHg inflation (all, *p* < 0.05).

**TABLE 2:** Effects of different intervention programs on other hemodynamic parameters.

	Conditions					Significance (*p* value)								
	P1	P2	P3	P4	P5	P1-P2-P3			P1-P2-P4			P1-P2-P5		
						P1-P2	P1-P3	P2-P3	P1-P2	P1-P4	P2-P4	P1-P2	P1-P5	P2-P5
BV (m/s)	0.21 ± 0.05	0.23 ± 0.06	0.28 ± 0.05	0.25 ± 0.07	0.23 ± 0.06	0.06	0.01	0.02	0.12	0.27	0.48	0.02	0.53	0.67
VD (cm)	0.43 ± 0.06	0.41 ± 0.03	0.41 ± 0.05	0.43 ± 0.04	0.41 ± 0.06	0.85	0.66	0.72	0.73	0.81	0.92	0.78	0.29	0.40
OSI	0.11 ± 0.06	0.07 ± 0.06	0.12 ± 0.04	0.15 ± 0.05	0.11 ± 0.05	**0.05**	0.76	0.03	0.20	0.23	0.05	0.01	0.66	0.17

Note: One-way repeated-measures ANOVA was used to compare the changes in each index between different intervention programs, and post hoc analysis (LSD) was used to assess the changes in BFV, VD, and OSI. P1, baseline; P2, HIIE; P3, HIIE + 100 mmHg; P4, HIIE + 80 mmHg; P5, HIIE + 60 mmHg; BV, blood velocity; VD, vessel diameter; OSI, oscillatory shear index; comparison of the index changes between the schemes: *p* < 0.05 means the difference is significant, *p* < 0.01 means the difference is very significant.

## 4 Discussion

Current studies on the effect of WSS-mediated exercise on vascular endothelial function have mainly been conducted in normal-weight individuals; thus, there may be BMI differences in the vascular response to exercise, which may limit the generalizability of the research findings (Hamburg et al., 2010; Slattery et al., 2016). In addition, cuffs with 60 mmHg inflation (Tinken et al., 2009) have mostly been used to promote WSS close to the baseline level following grip strength exercises, but the shear stress-mediated effects on vascular adaptation after HIIE in large systemic muscle groups remain unclear. Considering that HIIE is a high-intensity exercise, it may stimulate more blood flow than grip strength or other moderate-intensity aerobic exercises; thus, higher inflation might be required to reduce the shear stress from HIIE to close to that of the baseline level. Therefore, in this study, three different pressures of 60, 80, and 100 mmHg were applied during HIIE to determine the appropriate inflation that could maintain the WSS amplitude after HIIE near the baseline level (resting state). On this basis, we further explored the effect of WSS on vascular adaptation after acute HIIE exercise by manipulating WSS.

The main findings of this study were as follows: WSS was significantly higher in obese individuals after HIIE and HIIE with 100 mmHg inflation than that at baseline. There was no significant difference between post-HIIE and post-HIIE with 100 mmHg inflation. The RHI value of vascular endothelial function was significantly lower after HIIE with 100 mmHg inflation than after HIIE, but there was no significant difference compared to baseline. WSS after HIIE with 80 mmHg inflation and HIIE with 60 mmHg inflation showed no significant differences with HIIE alone. Vascular endothelial function was significantly lower than that after HIIE with 80 and 60 mmHg inflation than following HIIE. WSS and RHI were significantly higher after HIIE than at baseline, while there was no significant difference after HIIE with either 80 or 60 mmHg inflation. Collectively, these results suggested that HIIE could significantly improve WSS and vascular endothelial function. The HIIE intervention with 60 or 80 mmHg inflations, which represented sub-diastolic cuff inflation, might promote WSS near the baseline level. WSS might play a crucial role in HIIE for improving vascular endothelial function in obese men.

Similar to a previous study, cuff inflation was used to modulate blood flow and WSS during handgrip exercise (Green, 2009). The results of our study showed that WSS in obese individuals was significantly higher after HIIE than that at baseline, and could effectively improve vascular endothelial function. Moreover, WSS after HIIE with 100 mmHg inflation was significantly higher than WSS at baseline. To our surprise, WSS increased significantly after HIIE with 100 mmHg inflation, and, with regard to HIIE, there was no significant difference between HIIE and HIIE with 100 mmHg inflation. Moreover, the RHI value of vascular endothelial function after HIIE with 100 mmHg inflation was significantly lower than that after HIIE, with no significant difference compared to baseline. It can be speculated that the cuff with 100 mmHg inflation may be too high to impair the vascular endothelium and reduce vascular endothelial function, although it can effectively increase WSS (Zhou et al., 2014; Chistiakov et al., 2017; Tremblay and Pyke, 2018). In addition, another possible explanation was that the artery diameter decreased. Previous studies have shown that in a state of vascular stenosis, blood velocity increases, and the level of WSS increases significantly under pathological conditions (Sun and Liu, 2002; Barati et al., 2014). Therefore, we supposed that the inner diameter of the brachial artery might be narrowed during HIIE with 100 mmHg inflation. In addition, we also determined that blood velocity was significantly higher after HIIE with 100 mmHg inflation than following HIIE alone, which could thereby prompt a sharp increase in WSS, as observed in our study. Unfortunately, no significant difference in artery diameter was found after HIIE with 100 mmHg inflation, possibly due to insufficient intervention time. Previous studies have confirmed that changes in the adaptation of exercise in the arterial lumen may be related to the stimulation of shear stress by long-term exercise (Kumar et al., 2010; Sun HL, 2014).

At the same time, this study found that compared to HIIE, differences were not statistically significant in WSS following HIIE with 80 and 60 mmHg inflation. We also found that vascular endothelial function after HIIE with 80 and 60 mmHg cuff inflation was significantly lower than that following HIIE. Although WSS and vascular endothelial function-related parameters were not significantly different after HIIE among the three different inflations, based on the changes in WSS and vascular endothelial function observed after HIIE with 100 mmHg inflation, we speculated that 60 or 80 mmHg inflations, which were also below the values of diastolic blood pressure, might partly suppress or eliminate the increase in WSS by HIIE. This suppression would thereby neutralize the acute effects of HIIE exercise on brachial artery endothelial function, which is consistent with previous studies evaluating long-term training (Birk et al., 2012; Green et al., 2017).

In contrast to RHI, flow-mediated dilation (FMD) is widely used to evaluate the vascular endothelial function, but it still has several limitations, including complicated operation and lack of portability (Ras et al., 2013; Ramos et al., 2015; Bailey et al., 2017). RHI is a new indicator to evaluate vascular endothelial function (Hasin et al., 2015; Martini et al., 2020). Studies have confirmed that RHI and FMD have a linear correlation (Flammer et al., 2012; Poredos and Jezovnik, 2013). However, due to the technical requirements of the EndoPAT, it was impossible to test both limbs at the same time. Instead, the brachial artery of the nondominant limb was tested and the other limb was used for baseline correction. It should be noted that there may not be any significant differences in hemodynamic-related indicators or the structure of vascular function between bilateral limbs under normal circumstances. Therefore, in this study, WSS and vascular endothelial function in the baseline status were used as the controls for the effects of the cuff compression limb to demonstrate the WSS mechanism of HIIE in improving vascular endothelial function in obese men. Our study showed that compared with baseline, HIIE could effectively increase WSS and improve vascular endothelial function, although there was no significant difference in the changes in these indices after HIIE with 80 and 60 mmHg inflation, which was consistent with our hypothesis. Therefore, we speculate that the acute changes in WSS after HIIE could modulate the vascular endothelial function and that increased WSS may be an important stimulus for the acute enhancement of endothelial function after HIIE.

Many studies have confirmed that HIIE improves vascular function in obese people, which has been attributed mainly to traditional cardiovascular risk factors (Sawyer et al., 2016; De Lorenzo et al., 2018; Hovsepian et al., 2021; Mendelson et al., 2022). This study analyzed the possible mechanism through which HIIT improves vascular endothelial function from the perspective of WSS. Our results indicated that compared with baseline values, HIIE could significantly improve WSS and vascular endothelial function and that HIIE intervention with 60 or 80 mmHg inflations might promote WSS near the baseline level. Based on these findings, we speculate that the acute changes in WSS after HIIE and HIIE combined with different levels of pressure might modulate vascular endothelial function, and the increase in shear stress stimulation during HIIE intervention may be an important stimulus for the acute enhancement of endothelial function. Green et al. confirmed that shear stress could mediate vascular endothelial adaptations to exercise training in humans performing acute grip with 60 mmHg inflation when compared with healthy men for the training of small muscle groups, and these findings partly support our findings on WSS-mediated training adaptability (Thijssen et al., 2011; Green and Smith, 2018). Considering that HIIE is an exercise for large muscle groups, our study may have also indirectly explored the biomechanical mechanisms involved during the exercise of large muscle groups to improve vascular endothelial function.

Several limitations still exist in our study. First, to control for possible confounders of sex, only males were included in our sample. Therefore, it is unknown whether our findings could also apply to women. Because the vascular endothelial function and vascular reactivity are influenced by sex hormones, sex differences may exist in vascular responses to exercise, thereby limiting the generalizability of our results; thus further research needs to be conducted. Second, this study did not monitor the dynamic changes in WSS and vascular endothelial function during HIIE and HIIE with different inflations in real-time and only detected changes in blood flow and blood vessel-related indicators immediately after the interventions; thus there may be a certain time error. Third, this study did not analyze changes in relevant indicators in bilateral arms, although we determined resting-state data. Therefore, other confounding factors may be introduced, which could have a certain impact on the experimental results. Fourth, the main purpose of this study was to explore the mechanism by which WSS in HIIE improves vascular endothelial function in obese people. EndoPAT technology mainly evaluates vascular endothelial function by measuring the reactive hyperemia index of the upper extremities but not of the lower extremities. To ensure that WSS and vascular endothelial function were detected in the same arterial environment as much as possible, we chose the upper extremity as the main study focus. Nonetheless, the type of exercise used in this study was mainly HIIE with leg exercises, which could result in slight differences in the findings. Although previous studies have confirmed that exercise exerts systemic adaptive effects on blood vessels, this might represent a limitation of this study. Therefore, future studies can further explore the mechanism of WSS in improving vascular endothelial function on the main exercise limb in obese individuals after HIIE. Fifth, there was a 2-week washout period between each of the five tests in our experimental design, and any week-to-week variability might have influenced our results. Moreover, randomization of the intervention was not performed in our experimental design, which might also be a limitation in this study.

## 5 Conclusion

HIIE significantly improved WSS and vascular endothelial function, and HIIE with 60 or 80 mmHg inflation might promote WSS near the baseline level. The increase in WSS induced by HIIE may provide a powerful physiological stimulus for exercise training adaptation of vascular endothelial function in obese people. Future research should also explore mechanisms involved in WSS to improve vascular endothelial function in the main exercise limb in different sexes to further confirm the universality of the WSS mechanism in the adaptation of vascular endothelial function to exercise.

## Data Availability

The raw data supporting the conclusion of this article will be made available by the authors, without undue reservation.
